# Supplementary material for: Ex vivo assay for organ-specific cancer cell invasion
Source: J Biol Eng. 2026 Jun 23;20:142. doi: 10.1186/s13036-026-00719-9 (PMC13632008; doi:10.1186/s13036-026-00719-9)

Supplementary Figure 5

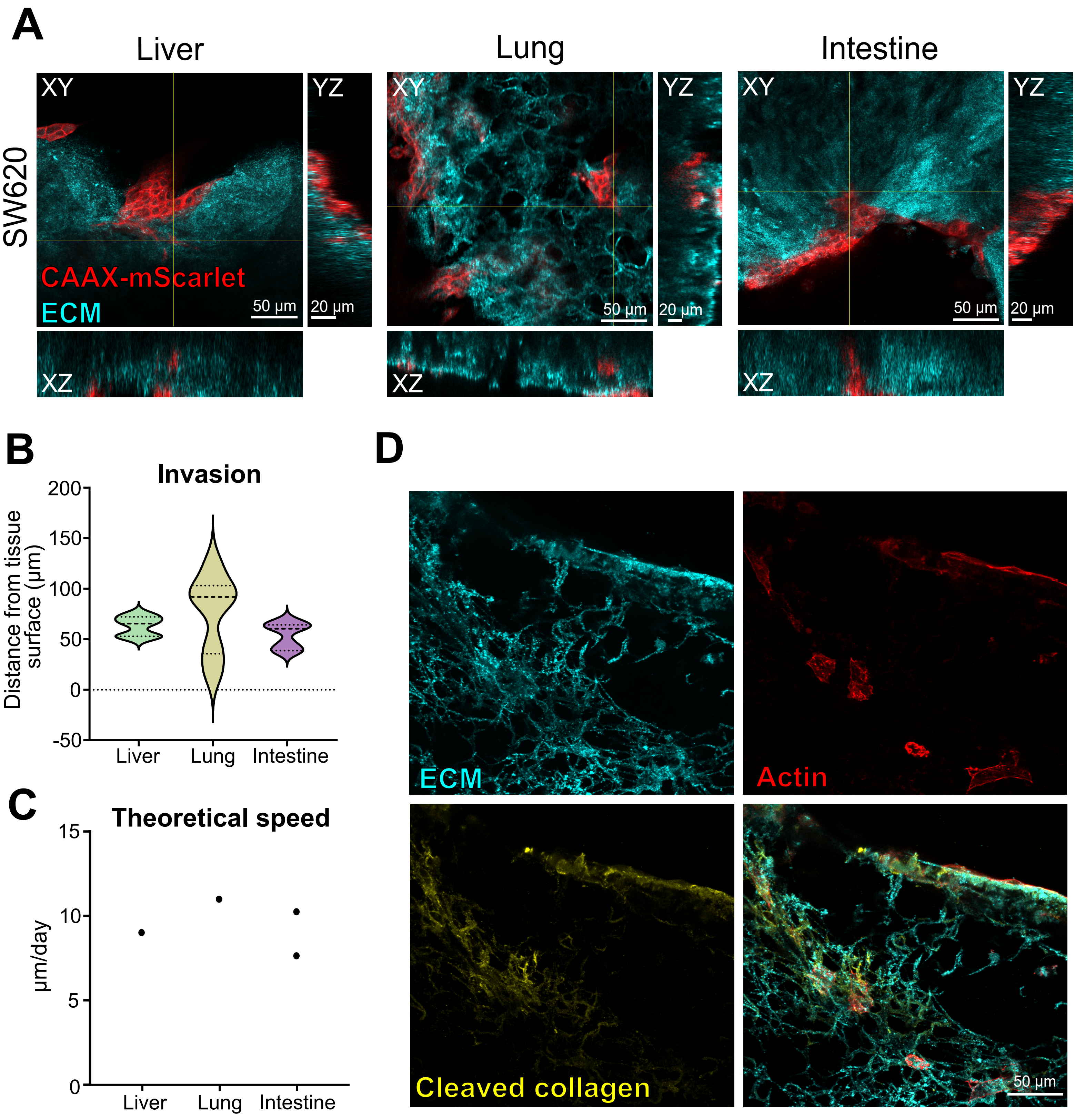

Supplement: Supplementary file 5 [file 13036_2026_719_MOESM5_ESM.pdf]
